# FGFR signaling and apoptotic regulation in cancer: links to immune evasion, therapeutic resistance, and treatment re-engagement

**DOI:** 10.3389/fimmu.2026.1953431

**Published:** 2026-09-17

**Authors:** Yinan Hu, Xinmiao Zhang, Wen Liu, Yuqing Wei, Jingli Xue, Xiaowen Ma, Yuan Cao, Peifeng Li

**Affiliations:** 1 Department of Pathology, The 960th Hospital of the People’s Liberation Army (PLA) Joint Logistic Support Force, Jinan, China; 2 College of Traditional Chinese Medicine, Shandong University of Traditional Chinese Medicine, Jinan, China; 3 Department of Pharmacy, The 960th Hospital of the People’s Liberation Army (PLA) Joint Logistic Support Force, Jinan, China; 4 Department of Basic Medical Sciences, The 960th Hospital of the People’s Liberation Army (PLA) Joint Logistic Support Force, Jinan, China

**Keywords:** apoptosis, FGFR signaling, immune evasion, mitochondrial apoptotic priming, targeted therapy, therapeutic resistance, tumor immunity

## Abstract

Fibroblast growth factor receptor (FGFR) signaling is a major oncogenic pathway in multiple cancer types and an important determinant of therapeutic response and resistance. However, its role in coordinating apoptotic susceptibility with tumor immune escape remains insufficiently integrated. This review summarizes how FGFR signaling regulates mitochondrial apoptosis through the RAS–RAF–MEK–ERK, PI3K–AKT–mTOR, PLCγ–PKC/Ca²^+^, and JAK–STAT pathways. These signaling networks converge on BCL-2 family proteins, BH3-only regulators, FOXO-dependent transcription, BAX/BAK activation, mitochondrial outer membrane permeabilization, cytochrome c release, and caspase activation. Non-canonical mechanisms involving redox homeostasis, metabolic stress, c-Myc regulation, and autophagy further determine whether FGFR inhibition produces transient adaptation or irreversible cell death. FGFR alterations may also shape the immune contexture of selected tumors by influencing inflammatory signaling, PD-L1 regulation, antigen-presentation pathways, tumor–stromal interactions, and susceptibility to immune-mediated elimination. These effects are strongly dependent on tumor lineage, genomic background, bypass receptor tyrosine kinase signaling, mitochondrial priming, and the local immune microenvironment. We further synthesize preclinical and emerging clinical evidence for combining FGFR-targeted therapies with immune checkpoint blockade and discuss the biomarkers and limitations that may determine therapeutic benefit. By linking apoptotic regulation, immune evasion, and therapeutic resistance, this review provides a framework for mechanism-based treatment strategies in FGFR-aberrant cancers.

## 1 Introduction

Fibroblast growth factor receptors (FGFRs) constitute a family of transmembrane receptor tyrosine kinases comprising four highly homologous members: FGFR1, FGFR2, FGFR3 and FGFR4. A canonical FGFR harbors an extracellular region with three immunoglobulin-like domains (D1–D3), a single transmembrane helix, and an intracellular tyrosine kinase domain. Upon binding to cognate fibroblast growth factor (FGF) ligands, FGFRs trigger downstream signaling cascades that regulate a wide spectrum of biological processes, including proliferation, differentiation, survival, migration, and metabolism (1). Under physiological conditions, FGFR signaling acts as a central hub for embryonic development, tissue homeostasis, and metabolic regulation (2), and its tight spatiotemporal control is essential for normal cellular and organismal function (3).

Constitutive or aberrant activation of FGFR signaling is a prevalent driver of human cancer. FGFR gene alterations occur in approximately 7.1% of solid tumors, encompassing gene amplifications, point mutations, and gene fusions/rearrangements (4, 5). These abnormalities frequently lead to ligand-independent or hyperactivated FGFR signaling, thereby promoting tumor initiation, progression, and therapeutic resistance in a tumor-type-specific manner. For instance, FGFR1 amplification is common in breast cancer and lung squamous cell carcinoma (6); FGFR2 fusions are frequently detected in intrahepatic cholangiocarcinoma (7, 8); FGFR3 mutations predominate in non-muscle-invasive bladder cancer (9); and FGF19-driven FGFR4 activation is implicated in hepatocellular carcinoma (10, 11).

In addition to driving tumor growth and survival, FGFR signaling is a key regulator of tumor cell apoptosis. Through canonical downstream pathways including RAS–RAF–MEK–ERK, PI3K–AKT, PLCγ–PKC, and JAK–STAT, FGFR signaling modulates core apoptotic regulators, such as BCL-2 family proteins, FOXO transcription factors, and components of the caspase cascade (12, 13). In many tumors, this signaling network enhances apoptotic resistance, enabling malignant cells to evade cytotoxic therapies, targeted agents, and immune-mediated elimination—thus contributing to two canonical hallmarks of cancer: resistance to cell death and immune evasion (14, 15).

The interplay between FGFR signaling and apoptosis remains incompletely defined. Current studies are often fragmented, focusing on individual downstream pathways rather than integrating them into a unified regulatory framework. Moreover, the impact of FGFR alterations on apoptosis varies markedly across tumor types, indicating strong dependence on tissue lineage, co-occurring molecular alterations, and network-level signaling context (16). Therefore, an integrated framework is urgently needed to clarify how FGFR signaling dictates apoptotic vulnerability.

In this review, we systematically dissect the molecular mechanisms by which FGFR signaling regulates apoptosis. Grounded in the molecular basis of FGFR signal transduction, we elucidate both canonical and non-canonical mechanisms governing cell apoptosis. We also discuss how tissue microenvironment, mutational background, and compensatory signaling shape apoptotic responses in FGFR-aberrant tumors. Finally, we address the challenges of restoring apoptotic responses in FGFR-targeted therapy, the emergence of drug resistance, and rational combination strategies, thereby providing novel insights into precision FGFR-targeted cancer treatment (17, 18).

In addition to sustaining tumor-cell survival, FGFR signaling may influence the interaction between malignant cells and the immune microenvironment. FGFR-dependent activation of ERK, AKT, and STAT pathways can affect inflammatory transcriptional programs, stress adaptation, immune-related ligand expression, and the ability of tumor cells to resist immune-mediated elimination. In selected tumor contexts, particularly FGFR3-altered urothelial carcinoma, FGFR abnormalities have been associated with relatively low-inflammatory phenotypes and distinct immune contextures. Moreover, emerging evidence suggests that FGFR inhibition may modify PD-L1 regulation and create opportunities for combination with immune checkpoint blockade. Nevertheless, these effects are not uniform across FGFR alterations or tumor lineages, and the relationship between FGFR dependency, apoptosis, and antitumor immunity remains incompletely defined. An integrated assessment is therefore needed to determine whether FGFR inhibition merely suppresses tumor-intrinsic survival signaling or also alters the immunological conditions required for durable therapeutic responses.

Although previous reviews have summarized FGFR signaling, FGFR-targeted therapies, or apoptosis regulation separately, the mechanistic connection between FGFR-driven oncogenic signaling and mitochondrial apoptotic control remains insufficiently integrated. In particular, how FGFR signaling maintains anti-apoptotic buffering, how FGFR inhibition lowers apoptotic restraint, and how resistance mechanisms restore survival signaling have not been systematically discussed within a unified translational framework. This review addresses this gap by integrating canonical FGFR signaling, non-canonical stress-adaptive mechanisms, tumor-type-specific FGFR alterations, tumor immune contexture, inhibitor responses, resistance mechanisms, and mechanism-based therapeutic strategies. Particular attention is given to the interface between FGFR-dependent apoptotic regulation and immune evasion, as well as to the emerging rationale and evidence for combining FGFR-targeted therapy with immune checkpoint blockade. By doing so, we aim to clarify how FGFR-dependent tumors may be therapeutically sensitized to apoptosis and immune-mediated elimination, and how rational combinations may convert transient therapeutic vulnerability into durable tumor control.

## 2 Molecular basis of FGFR signaling

### 2.1 Biochemical basis of FGFR activation: ligand binding, dimerization, and autophosphorylation

FGFR signaling is initiated by ligand binding, which induces receptor conformational rearrangement and dimerization. Most paracrine FGFs interact with heparan sulfate proteoglycans (HSPGs) on the cell surface to form a stable FGF–FGFR–HSPG ternary complex. This complex stabilizes receptor dimerization and promotes trans-autophosphorylation of the intracellular tyrosine kinase domains, thereby converting FGFR into an active signaling platform (19). FGFR autophosphorylation proceeds in a sequential and hierarchical manner. Phosphorylation first occurs at key tyrosine residues within the activation loop, causing a sharp increase in kinase activity, followed by phosphorylation of additional tyrosine residues that serve as docking sites for downstream signaling proteins (20). Taking FGFR1 as an example, its activation is a multi-step, highly ordered process in which distinct phosphorylation states determine not only catalytic activity but also the kinetics and selectivity of downstream effector recruitment (20). Through ligand binding, dimerization, and sequential autophosphorylation, FGFR translates extracellular growth factor cues into intracellular signaling outputs.

The biological outcome of FGFR activation depends not only on receptor phosphorylation status but also on the magnitude and duration of signaling. Transient versus sustained FGFR activation can elicit distinct transcriptional programs and cellular phenotypes, including differential sensitivity to apoptotic stimuli. These signaling dynamics are tightly regulated by receptor endocytosis, ubiquitination, and negative feedback mechanisms (21). Thus, understanding the molecular cascade of FGFR activation provides an essential framework for interpreting context-dependent apoptotic responses across tumor settings.

### 2.2 Major docking and adaptor proteins: FRS2, GRB2, GAB1, and PLCγ

Following autophosphorylation, phosphotyrosine residues in the FGFR intracellular domain act as docking sites for adaptor and effector proteins, routing signals into distinct downstream pathways. Among these, fibroblast growth factor receptor substrate 2 (FRS2) functions as the central scaffold for canonical FGFR signaling, and growth factor receptor bound protein 2 (GRB2) serves as a key adaptor linking the FGFR–FRS2 complex to RAS signaling. Upon phosphorylation by FGFR, FRS2 mediates both signal propagation and attenuation. On the one hand, phosphorylation motifs on FRS2 are recognized by the Src homology 2 (SH2) domain of GRB2. The SH3 domain of GRB2 recruits SOS to form the GRB2-SOS complex, which activates RAS and initiates the MAPK cascade. On the other hand, GRB2 mediates recruitment of the E3 ubiquitin ligase CBL, promoting ubiquitination and degradation of both the receptor and FRS2 to terminate signaling (22). This dual function is critical for apoptotic threshold regulation, as FRS2 not only amplifies signaling output but also restricts signal duration, thereby modulating cellular sensitivity to apoptotic stimuli. In addition, GRB2 facilitates recruitment of the docking protein GAB1, directing signaling toward the PI3K–AKT pathway. Although GAB1 is not FGFR-specific, it acts as both a signaling platform and an amplifier. Via multiple pYXXM motifs, GAB1 recruits the p85 regulatory subunit of PI3K, enhancing AKT activation and reinforcing pro-survival signaling (15). Thus, the GAB1/PI3K module represents a key branching node that biases FGFR output toward an anti-apoptotic state in many tumors.

Another critical branch parallel to the FRS2 scaffold system is the phospholipase C gamma (PLCγ) pathway. PLCγ is recruited via its SH2 domains to specific phosphotyrosine sites in the FGFR intracellular tail (e.g., FGFR1 pY766), and is activated in a phosphorylation-dependent manner. Activated PLCγ hydrolyzes phosphatidylinositol 4, 5-bisphosphate (PIP2) into diacylglycerol (DAG) and inositol 1, 4, 5-trisphosphate (IP3), triggering protein kinase C (PKC) activation and intracellular Ca^2+^ mobilization (23).

Because the PLCγ branch partially competes and interacts with the FRS2–GRB2 signaling complex at receptor docking and downstream signal routing, variable PLCγ engagement across cellular contexts may indirectly reshape the relative strength of MAPK and AKT signaling. Collectively, the FRS2–GRB2–GAB1 module primarily sustains proliferative and pro-survival signals, whereas the PLCγ branch more closely links FGFR signaling to Ca^2+^-dependent stress responses. Together, these branches establish the early signaling architecture of FGFR activation, determining signal routing and output bias, and thereby governing proliferative, survival, and stress-related cellular phenotypes.

## 3 Canonical downstream FGFR pathways and apoptosis regulation

Building on adaptor-mediated signal branching, activated FGFR transmits signals through several canonical downstream effector pathways, including RAS–RAF–MEK–ERK, PI3K–AKT, PLCγ–PKC, and in specific cellular contexts, JAK–STAT (**Figure 1**). These pathways do not function in isolation but collectively modulate cellular sensitivity to death stimuli by regulating mitochondrial homeostasis, transcriptional programs, metabolic adaptation, and stress responses. Therefore, understanding apoptosis regulation in FGFR-driven tumors requires not merely identifying the activation of individual pathways but rather clarifying how multiple downstream branches cooperate to set the apoptotic threshold. In the following sections, we discuss the pro-survival and pro-apoptotic effects of major canonical FGFR downstream pathways under defined conditions.

**Figure 1:**
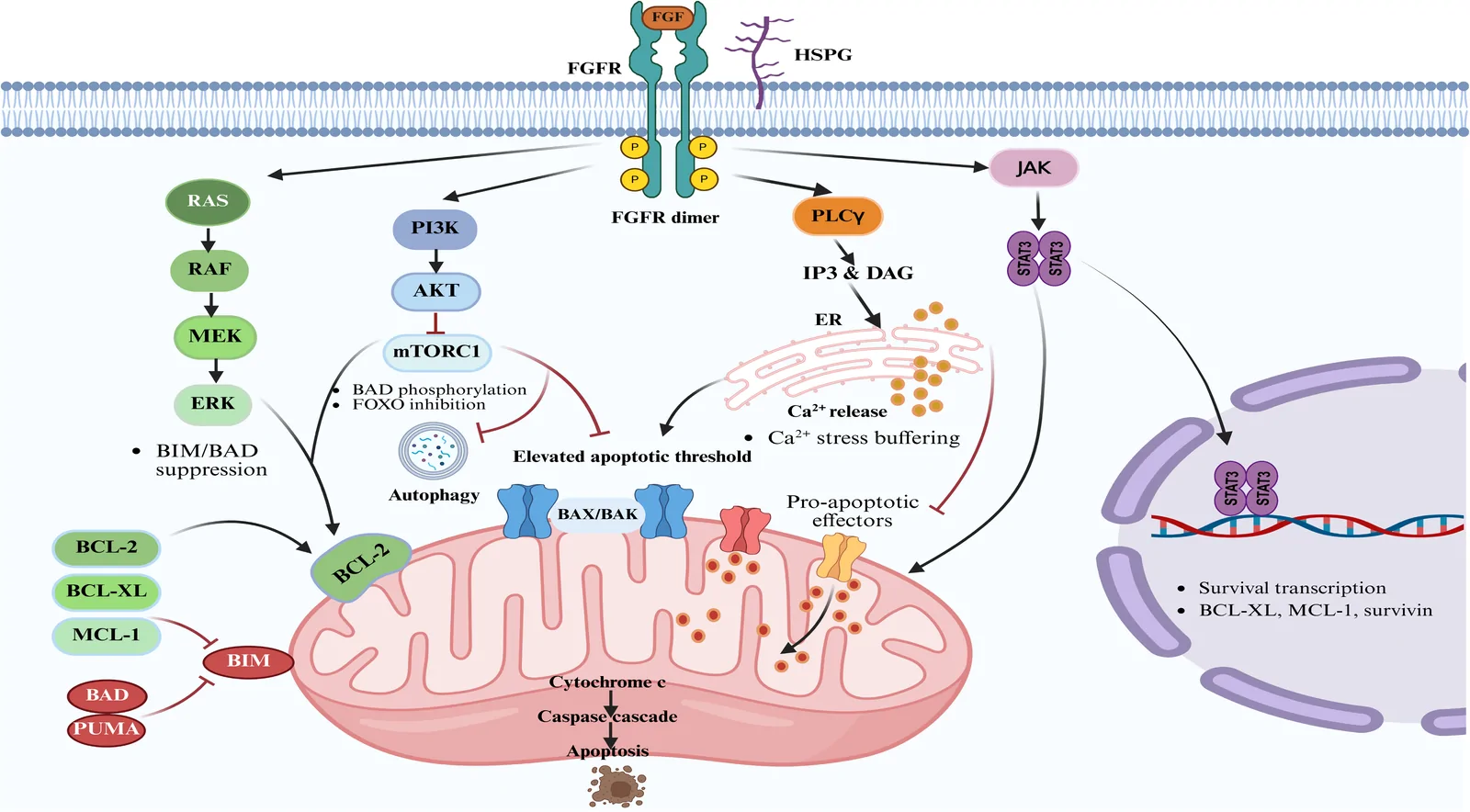
FGFR signaling suppresses apoptosis in cancer cells (Created in BioRender. Yinan, H. (2026) https://BioRender.com/0305wom). Activated FGFR triggers multiple downstream signaling branches, including RAS–RAF–MEK–ERK, PI3K–AKT–mTOR, PLCγ–PKC/Ca²^+^, and JAK–STAT pathways. These pathways converge on mitochondrial apoptotic machinery by suppressing pro-apoptotic BH3-only proteins, sustaining anti-apoptotic BCL-2 family members, promoting survival-associated transcription, and mitigating oxidative and organelle stress. Through this integrated network, FGFR signaling inhibits BAX/BAK activation and prevents mitochondrial outer membrane permeabilization, thereby limiting cytochrome c release, caspase activation, and the subsequent execution of apoptosis.

### 3.1 RAS–MAPK signaling and apoptosis regulation

In FGFR-driven tumors, the RAS–MAPK pathway serves as a central downstream node connecting receptor activation to cell fate reprogramming. Beyond driving proliferative programs, this pathway elevates the mitochondrial apoptotic threshold, conferring sustained survival advantages to tumor cells (24). Mechanistically, FGFR activation promotes recruitment of the GRB2–SOS complex through FRS2, facilitating GDP-GTP exchange on RAS and triggering sequential activation of RAF, MEK, and ERK.

Regarding apoptosis regulation, ERK signaling modulates core components of the mitochondrial apoptotic machinery via multiple downstream effectors (25). On the one hand, the ERK–RSK axis suppresses the pro-apoptotic activity of BH3-only proteins such as BIM and BAD, reducing BAX/BAK activation and limiting mitochondrial outer membrane permeabilization (MOMP). On the other hand, ERK signaling can maintain or enhance the stability of anti-apoptotic BCL-2 family proteins in certain tumor cells, further reinforcing apoptotic resistance (26, 27).

Notably, the apoptotic effects of ERK signaling are highly context-dependent. Transient or moderate activation generally supports cell survival, whereas sustained or excessive activation may become pro-apoptotic under oxidative stress, disrupted feedback control, or imbalanced stress signaling. For example, persistent ERK hyperactivation may promote cell death by amplifying reactive oxygen species (ROS), inducing stress-associated transcriptional programs, or perturbing dual-specificity phosphatase (DUSP) regulatory networks (28).

The RAS–MAPK pathway is not only a critical mechanism sustaining survival in FGFR-driven tumors but also a potential therapeutic vulnerability (26). Pharmacologic inhibition of MEK or ERK, as well as strategies to induce toxic “ERK overload” by disrupting feedback regulation, have been proposed to enhance anti-tumor efficacy (29). However, the success of such approaches likely depends on tumor molecular context and the balance between cytostatic and apoptotic responses.

### 3.2 PI3K–AKT signaling and apoptosis regulation

Among FGFR-activated downstream pathways, the PI3K–AKT axis represents one of the most robust barriers to apoptosis. By suppressing pro-apoptotic gene expression, preserving mitochondrial integrity, and promoting metabolic adaptation, this pathway establishes a durable anti-apoptotic state (30). It is typically engaged via GRB2-dependent recruitment of GAB1, which activates PI3K and efficiently couples FGFR signaling to sustained AKT activity (31).

AKT regulates apoptosis through multiple direct and indirect mechanisms. First, AKT phosphorylates BAD and promotes its sequestration by 14-3–3 proteins, relieving its inhibition of BCL-2 and BCL-XL and raising the MOMP threshold (32). Second, AKT phosphorylates FOXO transcription factors and drives their nuclear exclusion, suppressing expression of pro-apoptotic genes such as BIM and PUMA (33). In addition, AKT activates mTORC1, enhancing protein synthesis and metabolic fitness, enabling tumor cells to maintain anti-apoptotic protein levels under hypoxic or therapy-induced stress (34).

Despite its predominantly anti-apoptotic role, the contribution of the PI3K–AKT pathway remains highly context-dependent (35). Its importance is often amplified in tumors with GAB1 overexpression or co-occurring PI3K pathway alterations, whereas its relative influence may decline when other signaling branches dominate survival regulation (31).

Collectively, the PI3K–AKT axis frequently constitutes a critical bottleneck for apoptosis resistance in FGFR-driven tumors (36, 37). This may explain why FGFR inhibition alone is often insufficient to fully restore apoptotic capacity. In multiple preclinical models, combined targeting of FGFR and PI3K–AKT–mTOR signaling elicits stronger pro-apoptotic effects than FGFR inhibition alone (38), supporting this pathway as a major barrier to effective apoptotic reactivation.

### 3.3 PLCγ–PKC signaling and apoptosis regulation

Compared with the relatively consistent anti-apoptotic outputs of the ERK and AKT pathways, the PLCγ–PKC branch more tightly couples FGFR signaling to Ca^2+^ homeostasis, lipid-derived second messengers, and organelle stress responses. Consequently, its effects on apoptosis are more strongly context-dependent (39). Upon FGFR activation, PLCγ is directly recruited and phosphorylated, triggering hydrolysis of PIP2 into IP3 and DAG, which drive endoplasmic reticulum (ER) Ca^2+^ release and PKC activation, respectively (40).

In apoptotic regulation, Ca^2+^ acts as a critical second messenger governing ER–mitochondria crosstalk (41). Moderate Ca^2+^ signaling supports metabolic homeostasis and adaptive cell survival, whereas excessive or sustained Ca^2+^ release can trigger ER stress and mitochondrial Ca^2+^ overload, promoting MOMP, cytochrome c release, and caspase activation (42). Moreover, when protective pathways such as AKT are attenuated, Ca^2+^ dysregulation may more readily amplify apoptotic signaling via calpain activation or opening of the mitochondrial permeability transition pore (mPTP) (43).

Accordingly, the PLCγ–Ca^2+^/PKC axis can act either as an adaptive survival module or, under stress-induced disequilibrium, as a source of pro-death signaling (44). This functional duality underscores its importance in shaping cellular stress responses and determining whether FGFR signaling favors survival or apoptosis.

From a therapeutic perspective, the dual role of PLCγ–Ca²^+^ signaling may create a context-dependent vulnerability in FGFR-driven tumors. FGFR inhibition can weaken parallel pro-survival pathways such as AKT signaling, thereby reducing the capacity of tumor cells to buffer Ca²^+^-dependent stress. Under these conditions, additional perturbation of ER–mitochondrial Ca²^+^ transfer or enhancement of ER stress may further promote mitochondrial dysfunction and lower the apoptotic threshold. This provides a mechanistic rationale for combining FGFR-targeted therapy with approaches that disrupt Ca²^+^ homeostasis or intensify ER stress. However, because Ca²^+^ signaling is essential for normal cellular function, the therapeutic window and tumor selectivity of such combinations will require careful preclinical validation.

### 3.4 JAK–STAT signaling and apoptosis regulation

Whereas ERK and AKT primarily regulate apoptosis through rapid post-translational mechanisms, the STAT pathway mediates a more transcriptionally programmed mode of anti-apoptotic control downstream of FGFR. Its core role is to sustain pro-survival gene expression programs in tumor cells (45). Upon FGFR activation, STAT family members—particularly STAT3 and STAT5—are activated via direct or indirect mechanisms, followed by dimerization, nuclear translocation, and transcriptional induction of genes associated with survival and apoptosis resistance (46).

Among the STAT family members, STAT3 is particularly critical, as it directly upregulates anti-apoptotic factors including BCL-XL, MCL-1, and survivin, establishing a durable transcriptional barrier to apoptosis (47). Unlike the immediate effects mediated by ERK and AKT, STAT-dependent signaling maintains anti-apoptotic states over longer time scales by continuously reinforcing survival-associated transcriptional program (48).

As with other FGFR downstream branches, the role of STAT signaling is highly context-dependent. STAT3 generally acts as a pro-survival factor in tumors, whereas STAT1 may promote pro-apoptotic transcriptional programs under specific conditions (49). In addition, compensatory STAT3 activation is implicated as an important mechanism of resistance to FGFR inhibition, highlighting its role as both an effector pathway and an adaptive escape route (50).

Collectively, these findings provide a strong translational rationale for co-targeting the JAK–STAT pathway in FGFR-aberrant tumors (51). Inhibition of STAT3 may weaken anti-apoptotic transcriptional maintenance and enhance the pro-apoptotic effects of FGFR-targeted therapies (52, 53).

### 3.5 Network integration of pathway crosstalk in apoptosis control

Apoptosis regulation downstream of FGFR cannot be interpreted as a simple sum of isolated signaling branches. Instead, ERK, AKT, PLCγ, and STAT operate within an integrated signaling network that coordinately regulates mitochondrial homeostasis, transcriptional programs, metabolic adaptation, and organelle stress responses to determine apoptotic susceptibility (54).

Within this network, ERK and AKT cooperate at the post-translational level by jointly suppressing key pro-apoptotic regulators such as BAD and BIM (55). They also converge on mTORC1-dependent control of metabolism and autophagy, enhancing tumor cell fitness under persistent stress (56). STAT3 complements these relatively rapid mechanisms by converting transient pathway activation into sustained transcriptional support for anti-apoptotic genes, including BCL-XL, MCL-1, and survivin (50). In parallel, PLCγ-mediated Ca^2+^ signaling introduces an organelle stress dimension into this network. Under conditions of diminished AKT signaling or heightened cellular stress (57), Ca^2+^ imbalance can rapidly amplify apoptotic signaling via calpain activation or mPTP opening (58). Thus, tumor cell sensitivity to apoptotic stimuli is ultimately determined not by any single molecule or pathway, but by the integrated balance between pro-apoptotic and anti-apoptotic inputs across multiple FGFR downstream branches (59). This network-based perspective explains why FGFR signaling produces highly variable apoptotic outputs across tumor types and provides a mechanistic rationale for combination strategies that co-target multiple signaling branches to promote apoptosis (60).

Overall, canonical FGFR signaling regulates apoptosis through a dynamic and multilayered network rather than a single dominant axis. This framework also provides a conceptual basis for understanding non-canonical mechanisms of FGFR-mediated apoptosis regulation, including oxidative stress, autophagy, and metabolic vulnerability (61). Importantly, FGFR-driven apoptotic regulation may also influence tumor immune responses by shaping the cellular stress state and the susceptibility of tumor cells to immune-mediated elimination.

## 4 Non-canonical mechanisms of FGFR-mediated apoptosis regulation

FGFR signaling also modulates apoptosis through non-canonical mechanisms involving redox homeostasis, oncogenic transcriptional programs, and autophagic flux. Some of these processes (e.g., mitochondrial Ca^2+^ stress) functionally intersect with the PLCγ branch, but they also reflect broader roles of FGFR signaling in metabolic adaptation, stress buffering, and organelle homeostasis. Unlike canonical pathways, which primarily sustain survival via phosphorylation cascades and transcriptional control, these non-canonical mechanisms more directly reveal FGFR’s role in maintaining metabolic resilience and intracellular homeostasis. In the setting of pharmacologic FGFR inhibition, whether tumor cells remain in transient adaptation or cross the apoptotic threshold to undergo irreversible cell death may depend heavily on disruption of these processes.

Oxidative stress is one of the most consistent death-associated phenotypes following FGFR inhibition. In FGFR1-amplified or FGF-dependent lung cancer and multiple myeloma models, blockade of FGF/FGFR signaling induces ROS accumulation, exacerbates DNA damage, and increases caspase-dependent apoptosis, indicating that FGFR signaling in these tumors not only drives proliferation but also preserves redox homeostasis and buffers metabolic stress (62). Concomitantly, c-Myc downregulation is frequently an early molecular event after FGFR inhibition. Since c-Myc expression is partly sustained by the FGFR–ERK/PI3K axis, its decline impairs metabolic adaptation and stress tolerance, further amplifying ROS accumulation, mitochondrial injury, and apoptotic signaling (41). From this perspective, FGFR inhibition exposes not only dependence on canonical pro-survival pathways but also non-canonical vulnerabilities centered on ROS overload and c-Myc-associated metabolic fragility.

The second major layer of non-canonical regulation is the crosstalk between autophagy and apoptosis. First, during sustained FGFR activation PI3K–AKT–mTOR signaling commonly suppresses basal autophagic activity or reprograms autophagy as a survival-supporting process in FGFR-driven tumors (63). Second, following FGFR inhibition, relief of mTOR-mediated suppression can increase autophagic flux (64). At this early stage, enhanced autophagy may serve a cytoprotective role by clearing damaged mitochondria, limiting excessive ROS accumulation, and delaying apoptotic commitment. Third, when FGFR inhibition is prolonged and cellular stress exceeds the adaptive capacity of the autophagic machinery, autophagy may become dysregulated and shift toward a pro-death role. Under these conditions, Beclin-1-associated signaling, excessive organelle degradation, mitochondrial injury, and functional coupling with caspase activation may cooperate to promote irreversible apoptotic execution (65). Thus, the biological effect of autophagy after FGFR inhibition is temporally and contextually dependent, evolving from an early adaptive response toward a potential contributor to cell death when stress becomes persistent or overwhelming.

Collectively, these findings indicate that FGFR signaling sustains not only proliferative signaling but also a fragile equilibrium between metabolic adaptation and stress homeostasis. Once this balance is sufficiently disrupted, ROS accumulation, c-Myc downregulation, and autophagic imbalance may cooperate to drive tumor cells from protective adaptation toward lethal stress. These mechanisms expand the therapeutic implications of FGFR inhibition and highlight additional opportunities for combination strategies to promote apoptosis.

## 5 Tissue lineage, molecular context, and immune regulation shape FGFR-dependent therapeutic responses

### 5.1 Tissue lineage and molecular determinants of FGFR-dependent apoptotic responses

The type of FGFR alteration and its impact on apoptosis vary substantially across tumor types, underscoring a strong dependence on tissue lineage and molecular context. In lung cancer, particularly lung squamous cell carcinoma, FGFR1 amplification or overexpression is relatively common and may establish an FGF/FGFR-dependent survival program. In such models, blockade of FGF/FGFR signaling induces ROS accumulation, mitochondrial injury, and apoptosis, suggesting that cell death susceptibility depends partly on FGFR-mediated maintenance of redox homeostasis and metabolic adaptation (66). In cholangiocarcinoma, FGFR2 fusions are the most characteristic FGFR abnormality and often act as dominant oncogenic drivers, rendering tumors highly dependent on FGFR signaling. Even in this setting, however, apoptotic output remains shaped by ERK, STAT3, and bypass receptor tyrosine kinase signaling, which may explain the frequent emergence of adaptive resistance despite initial sensitivity to FGFR inhibitors (67). In urothelial carcinoma, FGFR3 alterations are common in non-muscle-invasive urothelial carcinoma and have been associated with proliferative advantage, relatively low-inflammatory phenotypes, and distinct tumor immune contextures. These features may influence sensitivity to immune checkpoint blockade, although the relationship remains dependent on disease stage, genomic background, and additional determinants of antitumor immunity. By contrast, in advanced disease, FGFR3 alterations may more readily translate into therapeutically meaningful apoptotic responses following FGFR inhibition (68). In breast cancer, especially hormone receptor-positive disease, FGFR1 amplification is a recurrent event that reinforces PI3K-AKT and ERK-dependent survival signaling and contributes to endocrine resistance. In this context, apoptotic resistance often manifests as a global reduction in treatment responsiveness rather than a single discrete molecular event (69).

These differences arise because FGFR alterations do not act in isolation but are embedded within tumor-specific molecular backgrounds and microenvironmental conditions (70). These microenvironmental determinants include not only ligand availability and stromal support but also inflammatory signaling, immune-cell infiltration, and the capacity of tumor cells to withstand immune-mediated cytotoxicity. Co-occurring genomic alterations (e.g., TP53, PIK3CA, RB1, or RAS pathway components) can markedly reshape cellular dependence on BH3-only proteins, cell-cycle checkpoints, or bypass survival pathways (71). Meanwhile, lineage-specific features and the local ligand environment influence the degree of FGFR dependency. Even with elevated FGFR expression or activity, only tumor cells that genuinely rely on FGF/FGFR signaling to maintain redox balance, metabolic fitness, or anti-apoptotic buffering are likely to undergo substantial apoptosis upon FGFR inhibition. In addition, microenvironmental cues and compensatory activation of parallel receptor tyrosine kinases further determine whether apoptosis can be sustainably re-engaged. Feedback activation of EGFR, ERBB (HER2), or MET, for example, may allow tumor cells to recover from transient stress and re-establish a pro-survival state (72). Accordingly, there is no uniform apoptotic pattern associated with FGFR alterations across cancers. Rather, apoptotic output is jointly determined by driver strength, parallel signaling network architecture, and microenvironmental constraints.

Notably, the strength of evidence linking FGFR alterations to specific apoptotic outcomes varies across tumor types. In tumors with concentrated driver dependence (e.g., FGFR2 fusion-positive cholangiocarcinoma), the connection between FGFR signaling and cell survival is well established. By contrast, in tumors characterized by FGFR1 amplification or multiple co-occurring oncogenic lesions, FGFR often represents only one important node within a broader survival network. In these settings, whether FGFR inhibition translates into substantial apoptosis likely depends on integrated factors including bypass receptor signaling, mitochondrial priming, and anti-apoptotic protein expression (**Table 1**).

**Table 1:** Tumor-type-specific FGFR alterations, apoptosis-related dependencies, and therapeutic strategies.

Tumor type	FGFR alteration	Involved pathways / dependencies	Effects of FGFR inhibition	FGFR inhibitor resistance mechanisms	Combination therapy strategies
Lung squamous cell carcinoma (43, 66)	FGFR1 amplification or overexpression	ERK/AKT survival signaling; redox buffering; metabolic adaptation; mitochondrial priming	ROS accumulation; mitochondrial injury; caspase-dependent apoptosis in susceptible models	Bypass RTK activation; incomplete MAPK/PI3K–AKT suppression; variable mitochondrial priming	FGFR inhibitor plus MAPK-pathway inhibition, PI3K/AKT/mTOR blockade, or stress-enhancing strategies
Intrahepatic cholangiocarcinoma (7, 13, 67, 80, 83)	FGFR2 fusion or rearrangement	Strong driver dependence; ERK/STAT3 signaling; bypass RTKs; network plasticity	Survival signaling reduction; apoptosis facilitation; limited durable cell death due to adaptive rewiring	Secondary FGFR kinase-domain mutations; ERK reactivation; STAT3 compensation; bypass RTK activation; convergent MAPK alterations	FGFR inhibitor plus MEK/ERK inhibition, STAT3-targeted approaches, BH3 mimetics, or next-generation FGFR inhibitors/degraders
Urothelial carcinoma (68, 75, 79)	FGFR3 mutation, fusion, or activation	Disease-stage dependence; lineage state; immune contexture; coexisting survival pathways	Tumor regression and apoptosis-related effects in selected advanced disease; heterogeneous apoptotic dependence	Adaptive signaling reprogramming; bypass RTKs; partial downstream reactivation; persistent anti-apoptotic buffering	Context-specific co-targeting of PI3K/AKT/mTOR, MAPK, or BCL-2 family-mediated survival pathways
Hormone receptor-positive breast cancer (6, 69, 87)	FGFR1 amplification	ERK/PI3K survival signaling; mitochondrial anti-apoptotic buffering; endocrine resistance	Limited apoptosis with FGFR inhibition alone; reduced endocrine or treatment responsiveness predominates	PI3K–AKT rebound; persistent ERK signaling; endocrine crosstalk; broader network compensation	FGFR inhibitor plus endocrine therapy, PI3K/AKT/mTOR inhibition, MAPK-pathway blockade, FGFR degrader, or apoptosis-sensitizing strategies
Hepatocellular carcinoma (10, 11, 85)	FGF19–FGFR4 axis activation	Lineage-specific FGFR4 signaling; metabolic regulation; downstream pathway addiction	Context-dependent apoptosis in FGFR4-dependent settings; dependence on pathway addiction and survival circuitry	Incomplete pathway dependence; compensatory RTK signaling; PI3K/mTOR reactivation; downstream survival restoration	FGFR4-axis inhibition plus downstream pathway inhibitors or apoptosis-sensitizing strategies

AKT, protein kinase B; BCL-2, B-cell lymphoma 2; BH3, BCL-2 homology 3; ERK, extracellular signal-regulated kinase; FGFR, fibroblast growth factor receptor; MAPK, mitogen-activated protein kinase; MCL-1, myeloid cell leukemia 1; mTOR, mechanistic target of rapamycin; PI3K, phosphoinositide 3-kinase; ROS, reactive oxygen species; RTK, receptor tyrosine kinase; STAT3, signal transducer and activator of transcription 3.

Representative references are listed after each tumor type. Apoptotic responses to FGFR inhibition are context-dependent and influenced by tumor lineage, genomic background, mitochondrial priming, and compensatory survival signaling.

### 5.2 FGFR alterations and tumor immune contexture

Beyond tumor-intrinsic survival regulation, FGFR alterations may influence the composition and functional state of the tumor immune microenvironment. This relationship has been most extensively investigated in urothelial carcinoma, where FGFR3 alterations are enriched in luminal-papillary tumors and have been associated with relatively low-inflammatory or non-T-cell-inflamed phenotypes (73). Transcriptomic studies have identified an inverse association between FGFR3 activity and CD8A expression, together with reduced interferon-related signaling and T-cell infiltration in FGFR3-associated tumors (74). These findings suggest that FGFR3-driven tumor biology may contribute to immune exclusion, although whether this represents a direct consequence of FGFR3 signaling or the broader molecular characteristics of luminal tumors remains incompletely resolved.

Recent mechanistic studies further indicate that FGFR3 signaling may actively shape an immunologically inactive tumor microenvironment. FGFR3-altered tumors exhibit reduced immune-cell infiltration, impaired T-cell activation, and increased immunosuppressive myeloid-cell features (75). In addition, FGFR3-driven metabolic reprogramming may influence macrophage function, antigen presentation, and cytotoxic lymphocyte recruitment, suggesting that FGFR signaling regulates antitumor immunity through both tumor-cell-intrinsic and microenvironmental mechanisms (76).

FGFR signaling may also intersect with immune regulation through interferon and immune-checkpoint pathways. In bladder cancer models, FGFR inhibition promotes autophagic degradation of IFN-γ-induced PD-L1, linking FGFR activity, autophagy, and immune-checkpoint regulation (18). However, changes in PD-L1 expression after FGFR inhibition should not be interpreted as evidence that FGFR blockade will universally enhance antitumor immunity. The immunological consequences of FGFR inhibition are likely influenced by FGFR alteration type, tumor lineage, baseline interferon responsiveness, stromal composition, and immune-cell states.

### 5.3 Context-dependent effects of FGFR signaling on antitumor immunity

Importantly, the immune-cold phenotype associated with FGFR3 alterations does not necessarily translate into uniform resistance to immune checkpoint blockade. Clinical studies have reported variable associations between FGFR3 status and immunotherapy outcomes, suggesting that FGFR alteration alone is insufficient to define immune responsiveness (77–79). This discrepancy highlights the importance of distinguishing oncogenic genotype from functional immune state. Although FGFR alterations may favor immune-excluded phenotypes in selected tumor contexts, the ultimate immune response is determined by multiple additional factors, including tumor mutational burden, antigenicity, interferon signaling, T-cell activation, myeloid-cell composition, and stromal programs.

From the perspective of apoptosis, these determinants may influence whether tumor cells surviving FGFR inhibition can subsequently undergo immune-mediated elimination. Therefore, the FGFR–apoptosis–immunity interface should be viewed as a context-dependent network rather than a linear pathway. Future studies integrating FGFR molecular profiling with functional immune characterization will be essential for identifying patients most likely to benefit from FGFR-targeted immunomodulatory strategies.

## 6 FGFR-targeted therapy and reactivation of apoptosis

### 6.1 Clinical efficacy and resistance mechanisms of FGFR inhibitor monotherapy

The therapeutic value of FGFR-targeted strategies extends beyond suppressing tumor cell proliferation. More critically, these approaches disrupt the survival dependency imposed by aberrant FGFR signaling and shift tumor cells toward an apoptosis-prone state. In FGFR-driven tumors, ERK, PI3K–AKT, STAT signaling, and redox homeostasis collectively form a robust anti-apoptotic network (**Figure 2**). By restraining BH3-only proteins, reinforcing BCL-2 family-mediated survival buffering, and preserving mitochondrial integrity, this network limits cellular responsiveness to death-inducing stimuli. Effective FGFR inhibition destabilizes this protective state, leading to partial restoration of pro-apoptotic signaling, attenuation of anti-apoptotic defenses, and reduced tolerance to cellular stress. Thus, the biological consequence of FGFR blockade is not merely interruption of oncogenic signaling but re-sensitization of tumor cells to apoptosis (24).

**Figure 2:**
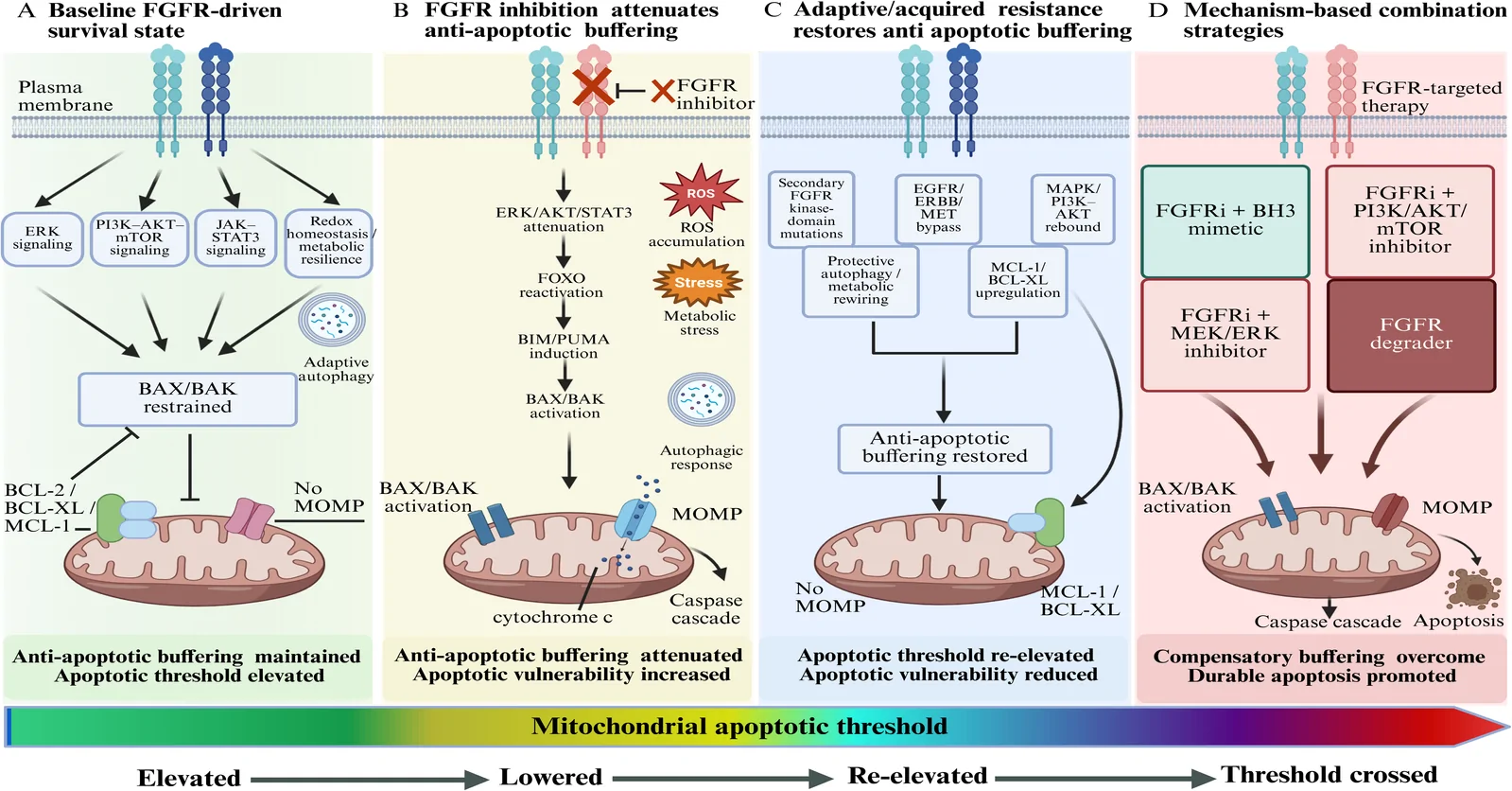
FGFR-targeted therapy and reactivation of apoptosis in cancer cells (Created in BioRender. Yinan, H. (2026) https://BioRender.com/h1zu4nr). **(A)** Baseline FGFR-driven survival state. **(B)** FGFR inhibition attenuates anti-apoptotic buffering. **(C)** Adaptive/acquired resistance restores anti-apoptotic buffering. **(D)** Mechanism-based combination strategies. FGFR inhibition attenuates anti-apoptotic buffering by reducing ERK, AKT, and STAT3 survival signaling, reactivating FOXO-dependent pro-apoptotic programs, inducing BIM/PUMA expression, and promoting BAX/BAK activation, ROS accumulation, metabolic stress, and autophagic responses. Adaptive or acquired resistance can restore anti-apoptotic buffering through secondary FGFR kinase-domain mutations, EGFR/ERBB/MET bypass signaling, MAPK/PI3K–AKT rebound, MCL-1/BCL-XL upregulation, and protective autophagy/metabolic rewiring. Mechanism-based combination strategies may overcome compensatory survival buffering and convert transient apoptotic vulnerability into durable cell death.

Clinically, small-molecule tyrosine kinase inhibitors (TKIs) remain the most established modality for FGFR targeting. Pemigatinib, a selective FGFR1–3 inhibitor, achieved an objective response rate of 35.5% in the FIGHT-202 study in previously treated patients with FGFR2 fusion- or rearrangement-positive cholangiocarcinoma (80), and long-term follow-up confirmed durable response in a subset of patients (81). Erdafitinib provided robust randomized evidence in urothelial carcinoma with FGFR2/3 alterations, improving overall survival compared with chemotherapy in the phase III THOR trial (82). Collectively, these studies demonstrate that FGFR inhibition can not only constrain tumor growth but also weaken survival signaling sufficiently to facilitate apoptotic cell death in selected FGFR-dependent settings.

Nevertheless, FGFR inhibitors as monotherapy often fail to elicit durable and robust apoptotic responses. This limitation reflects that FGFR—while important as an oncogene—is rarely the sole determinant of tumor cell survival. Following FGFR blockade, tumor cells may maintain viability via bypass activation of other RTKs, reactivation of downstream MAPK or PI3K–AKT–mTOR signaling, or compensatory upregulation of anti-apoptotic proteins. Acquired resistance can thus be defined as survival network reprogramming. Common mechanisms include secondary mutations in the FGFR kinase domain, activation of bypass pathways mediated by EGFR, ERBB family members, or MET, restoration of MAPK or PI3K–AKT–mTOR signaling, and adaptive increases in anti-apoptotic proteins such as MCL-1 and BCL-XL in certain models (83). In this context, FGFR inhibition should be viewed primarily as a means to lower the apoptotic threshold rather than a sufficient trigger of irreversible cell death. Efficient apoptosis execution depends on simultaneous suppression of these alternative survival mechanisms.

### 6.2 Apoptosis-sensitizing combinations and co-targeting of survival pathways

This framework provides a mechanistic rationale for combination therapy to overcome barriers to apoptosis reactivation. In tumors heavily reliant on mitochondrial anti-apoptotic buffering, combining FGFR inhibitors with BH3 mimetics may more effectively dismantle BCL-2 family protection and promote MOMP (84). In models characterized by compensatory PI3K–AKT–mTOR axis activation after FGFR inhibition, co-targeting this pathway with PI3K, AKT, or mTOR inhibitors may help sustain a reduced survival threshold. Similarly, in tumors with rebound MAPK activation, combinations of FGFR inhibitors with MEK or ERK inhibitors are supported by a clear mechanistic rationale (85). Although most of these strategies remain preclinical or early translational, they align with the concept that therapeutic success depends on converting reduced apoptotic restraint into effective cell death execution (83).

Beyond TKIs, antibody-based and receptor degradation strategies offer complementary approaches to target aberrant FGFR signaling. The FGFR3-directed monoclonal antibody vofatamab (B-701) has undergone early clinical evaluation in urothelial carcinoma, although current evidence supports its role primarily as an investigational or adjunctive approach rather than an established therapeutic standard (86). In contrast, targeted protein degradation strategies are mechanistically appealing, as they eliminate the receptor itself rather than merely inhibiting kinase activity. This may enable more complete abrogation of downstream signaling through FRS2, AKT, and ERK. Recent preclinical work shows that the FGFR1/2 degrader DGY-09–192 achieves more profound pathway suppression than conventional inhibitors in ER-positive breast cancer models harboring FGFR1/2 alterations, while enhancing sensitivity to endocrine therapy. Although these approaches remain preclinical, they suggest that receptor degradation may represent a more effective route to apoptosis reactivation in selected molecular settings (87).

### 6.3 FGFR inhibition combined with immune checkpoint blockade

The relationship between FGFR signaling and tumor immune regulation provides a rationale for combining FGFR-targeted therapy with immune checkpoint blockade. This strategy is particularly relevant in tumors such as urothelial carcinoma, where FGFR alterations are associated with distinct immune phenotypes. FGFR inhibition may enhance immunotherapy through multiple mechanisms, including reducing tumor-cell survival capacity, altering PD-L1 regulation, influencing antigen-presentation pathways, and modifying tumor–immune interactions. However, the immunological consequences of FGFR inhibition are highly context-dependent and cannot be assumed to uniformly enhance antitumor immunity.

Preclinical studies indicate that FGFR blockade may influence immune-cell function and immune-regulatory pathways, although the direction of these effects varies according to tumor lineage, FGFR alteration type, baseline immune state, and the molecular mechanisms controlling immune-checkpoint regulation (88–90). Early clinical studies, including FORT-2 and NORSE, have demonstrated the feasibility and preliminary clinical activity of combining FGFR inhibitors with immune checkpoint blockade in selected patient populations (91, 92). However, these studies remain exploratory and have not yet established whether combination therapy is universally superior to FGFR-targeted monotherapy.

Therefore, FGFR alteration status alone is unlikely to be sufficient for patient selection. Future studies should integrate FGFR pathway dependency with biomarkers reflecting immune competence, including PD-L1 regulation, interferon-related signaling, antigen-presentation capacity, immune-cell composition, and treatment-induced changes in the tumor microenvironment. Importantly, FGFR inhibitor-induced apoptosis should also be distinguished from immunogenic cell death, because lowering the apoptotic threshold does not necessarily generate sufficient inflammatory signaling, antigen presentation, or durable T-cell activation.

### 6.4 Biomarker-guided combination strategies

The future success of FGFR-targeted therapy will likely depend on moving beyond static genomic classification toward integrated biomarker-guided treatment strategies. Relevant biomarkers may include FGFR alteration type, pathway dependency, bypass receptor activation, MAPK or PI3K–AKT pathway rebound, BCL-2 family dependence, mitochondrial priming, autophagic adaptation, and immune contexture. Integrating these tumor-intrinsic and immune-related features may help identify patients most likely to achieve durable apoptosis reactivation and benefit from rational FGFR-based combination therapies. Overall, the capacity of FGFR-targeted therapy to induce apoptosis depends on how effectively it relieves sustained suppression of pro-apoptotic programs by ERK, AKT, STAT, and related survival pathways. Its therapeutic efficacy, however, is constrained by tumor cells’ ability to reconstruct pro-survival signaling via adaptive and acquired resistance mechanisms. Future treatment strategies will likely be most effective when guided by precise identification of dominant resistance drivers and key apoptotic bottlenecks. In this setting, rational combinations of FGFR inhibitors with BH3 mimetics, PI3K/AKT/mTOR inhibitors, MAPK pathway inhibitors, receptor-degrading technologies, or immunotherapies may offer the greatest potential to convert transient signal suppression into durable apoptosis reactivation. Therefore, future FGFR-directed strategies should be guided by biomarkers that capture both FGFR dependency and apoptotic readiness, including FGFR alteration type, bypass RTK activation, MAPK/PI3K pathway rebound, BCL-2 family dependence, mitochondrial priming, and autophagic adaptation.

## 7 Discussion and future perspectives

The FGFR signaling pathway is biologically pivotal in cancer, not only because it promotes proliferation and sustains oncogenic signaling but also because it establishes and maintains the apoptotic threshold of tumor cells. This function is achieved through the coordinated action of canonical downstream pathways and non-canonical stress-adaptive mechanisms. The apoptotic output associated with FGFR alterations is not fixed; rather, it is dynamically shaped by FGFR aberration type, downstream signaling network architecture, co-mutational landscape, lineage-specific characteristics, and the compensatory capacity of the tumor microenvironment. Importantly, these contextual factors may also influence the immune consequences of FGFR alterations, highlighting the need to integrate apoptotic regulation with tumor immune contexture. Accordingly, FGFR may act as a dominant survival dependency in some tumors, whereas in others it functions as part of a broader pro-survival network. Understanding the FGFR–apoptosis axis therefore requires more than cataloguing FGFR-regulated death processes; it demands defining the molecular contexts in which FGFR becomes a decisive regulator of cell fate.

The therapeutic value of FGFR inhibition lies not only in suppressing tumor growth but in converting FGFR dependency into effective and durable apoptotic execution. Current evidence indicates that FGFR-targeted therapies can lower the apoptotic threshold by relieving survival signaling mediated by ERK, AKT, and STAT, while simultaneously exposing vulnerabilities related to oxidative stress, metabolic imbalance, and organelle dysfunction. However, the durability of these effects is often limited by secondary kinase-domain mutations, compensatory activation of bypass RTKs, downstream pathway reactivation, and adaptive upregulation of anti-apoptotic proteins. Thus, the key clinical question is no longer simply whether FGFR can be targeted, but which mechanisms prevent tumor cells from undergoing irreversible apoptosis after FGFR inhibition.

Emerging evidence further suggests that FGFR signaling may connect tumor-intrinsic apoptotic resistance with immune evasion. In selected tumor contexts, particularly FGFR3-altered urothelial carcinoma, FGFR abnormalities have been associated with relatively low-inflammatory phenotypes and distinct immune contextures. FGFR inhibition may also influence IFN-γ-induced PD-L1 regulation, autophagic processing, tumor–stromal interactions, and susceptibility to immune-mediated elimination. Nevertheless, these immunological effects are unlikely to be uniform across tumor types or FGFR alterations. It remains unclear whether the immune phenotypes observed in FGFR-altered tumors are directly driven by FGFR signaling or instead reflect the lineage, genomic background, stromal composition, and treatment history in which these alterations arise. Moreover, apoptosis induced by FGFR inhibition should not automatically be interpreted as immunogenic cell death, because conventional apoptotic clearance may occur without sufficient inflammatory signaling, antigen presentation, or durable T-cell activation. Future studies should therefore distinguish FGFR genotype from functional immune phenotype and determine which immunological changes are directly driven by FGFR signaling and which are reversible through FGFR inhibition.Future therapeutic development should move beyond a static view of FGFR alteration status and incorporate dynamic biomarkers of FGFR dependency. Such biomarkers may include FGFR alteration type, pathway activity, bypass RTK activation, MAPK or PI3K–AKT pathway rebound, BCL-2 family dependence, mitochondrial priming, autophagic adaptation. Mechanism-based combinations of FGFR inhibitors with BH3 mimetics, PI3K/AKT/mTOR inhibitors, MEK or ERK inhibitors, receptor-degrading approaches, or immune checkpoint blockade may offer the greatest potential to convert transient signaling inhibition into durable tumor control. However, these combinations should be selected according to the dominant resistance mechanism and the biological context of each tumor rather than applied uniformly to all FGFR-aberrant cancers. A deeper understanding of how FGFR signaling intersects with mitochondrial apoptotic priming, immune contexture, and signaling-network plasticity will be essential for developing more durable and biomarker-guided treatment strategies for FGFR-driven malignancies.
